# Sweetened Beverages, Coffee, and Tea and Depression Risk among Older US Adults

**DOI:** 10.1371/journal.pone.0094715

**Published:** 2014-04-17

**Authors:** Xuguang Guo, Yikyung Park, Neal D. Freedman, Rashmi Sinha, Albert R. Hollenbeck, Aaron Blair, Honglei Chen

**Affiliations:** 1 Department of Health Studies, Westat Inc., Research Triangle Park, North Carolina, United States of America; 2 Nutritional Epidemiology Branch, National Cancer Institute, Rockville, Maryland, United States of America; 3 AARP, Washington DC, United States of America; 4 Occupational and Environmental Epidemiology Branch, National Cancer Institute, Rockville, Maryland, United States of America; 5 Epidemiology Branch, National Institute of Environmental Health Sciences, Research Triangle Park, North Carolina, United States of America; National Center of Neurology and Psychiatry, Japan

## Abstract

Sweetened beverages, coffee, and tea are the most consumed non-alcoholic beverages and may have important health consequences. We prospectively evaluated the consumption of various types of beverages assessed in 1995–1996 in relation to self-reported depression diagnosis after 2000 among 263,923 participants of the NIH-AARP Diet and Health Study. Odds ratios (OR) and 95% confidence intervals (CI) were derived from multivariate logistic regressions. The OR (95% CI) comparing ≥4 cans/cups per day with none were 1.30 (95%CI: 1.17–1.44) for soft drinks, 1.38 (1.15–1.65) for fruit drinks, and 0.91 (0.84–0.98) for coffee (all *P* for trend<0.0001). Null associations were observed for iced-tea and hot tea. In stratified analyses by drinkers of primarily diet versus regular beverages, the ORs were 1.31 (1.16–1.47) for diet versus 1.22 (1.03–1.45) for regular soft drinks, 1.51 (1.18–1.92) for diet versus 1.08 (0.79–1.46) for regular fruit drinks, and 1.25 (1.10–1.41) for diet versus 0.94 (0.83–1.08) for regular sweetened iced-tea. Finally, compared to nondrinkers, drinking coffee or tea without any sweetener was associated with a lower risk for depression, adding artificial sweeteners, but not sugar or honey, was associated with higher risks. Frequent consumption of sweetened beverages, especially diet drinks, may increase depression risk among older adults, whereas coffee consumption may lower the risk.

## Introduction

Sweetened beverages such as soft drinks, along with coffee and tea, are the most consumed non-alcoholic beverages throughout the world, and have substantial public health consequences [1]. Regular sweetened beverages contain large amounts of sugar and may have contributed to the obesity and diabetes epidemic in Western countries [2], [3], [4], [5]. The recent trend of increasing diet drink consumption is also of possible concern. These beverages may contain artificial sweeteners such as aspartame and saccharin; although there is controversy, potential adverse effects of these substances have been suspected [6], [7]. On the other hand, drinking coffee and tea has been linked to a lower risk for diabetes [8]. In addition, caffeine and its major metabolites are well-documented brain stimulants and there is growing evidence for potential benefits of caffeine towards brain health [9], including lower risk for Parkinson's disease [10], [11], [12] and dementia [13].

The potential effects of drinking these beverages on depression, however, are largely unknown. In several cross-sectional studies, frequent drinking of sweetened beverages was associated with higher prevalence of depression, suicidal thoughts and acts, and other mental stress [14], [15], [16]. However, this relationship could be bi-directional and cross-sectional analyses cannot rule out reverse causality. A few prospective studies suggest that coffee drinking lowers the risk for suicide [17], [18], [19] and depression [20], [21]. We evaluated relationships between consumption of sweetened beverages, coffee, or tea and depression among 263,923 participants of the prospective NIH-AARP Diet and Health Study.

## Materials and Methods

### Study population and case identification

The NIH-AARP Diet and Health study (http://dietandhealth.cancer.gov/), a prospective cohort, was established in 1995–1996 by the National Cancer Institute to evaluate the etiology of cancer and other chronic diseases [22]. The cohort is composed of 566,398 AARP (previously known as American Association of Retired Persons) members (ages 50–71) from six US states and two metropolitan areas who satisfactorily completed a comprehensive survey on diet and lifestyle. A follow-up questionnaire was sent to original cohort participants in 2004–2006 to update information on lifestyle and to ascertain the occurrence of major chronic diseases, including depression. A total of 318,257 participants (187,496 men and 130,761 women) returned the follow-up questionnaire and were eligible for the present analyses. On the follow-up questionnaire, participants were asked whether they had ever been diagnosed by a doctor as having depression and if so the year of first diagnosis in the following categories: “before 1985”, “1985–1994”, “1995–1999”, or “2000 to present”. A total of 41,074 participants reported a diagnosis of depression on the follow-up questionnaire, 21,370 before 1995 (baseline survey), 8,219 between 1995 and 1999, and 11,485 since 2000. We limited the present analyses to depression cases with a first diagnosis since 2000 in order to reduce the potential impact of reverse causation, as dietary changes due to depression could possibly affect our results. Of the 277,186 participants not reporting a diagnosis of depression, we excluded 20,893 participants with missing or inconsistent information on depression diagnosis. We also excluded 3,678 participants without a depression diagnosis and 174 cases because of missing or inconsistent data on the exposures of interest. After these exclusions, the primary analyses included a total of 11,311 depression cases and 252,612 participants without depression. Participants consented to the study by returning survey questionnaires. The study protocol was approved by the Institutional Review Board of the National Institute of Environmental Health Sciences and the Special Studies Institutional Review Board of the National Cancer Institute.

### Exposure assessment

The cohort's baseline survey included a food frequency questionnaire that assessed consumption frequency and portion size of 124 food items in the past year, including “soft drinks, soda, pop (diet or regular)” (referred as soft drinks), “drinks such as Hi-C, lemonade, Kool-Aid” (referred as fruit drinks), “coffee”, “hot tea” and “iced-tea”. Ten consumption frequencies were allowed for each drink, ranging from “never” to “≥6 times/day” for soft and fruit drinks, or from “none” to “6+cups/day” for coffee and teas. Three portion sizes were provided for consumption of soft drinks (<10 oz or <1 can, 10–12 oz or 1 can, >12 oz or >1 can) and fruit drinks (<1 cup, 1–1.5 cups, and >1.5 cups). We converted consumptions of soft and fruit drinks into cans per day based on reported frequency and portion size. For soft drinks, fruit drinks, and sweetened iced-tea, we further asked whether participants drank “sugar-free (diet)” or “regular-calorie” types for more than half of the time. Drinkers were further classified as drinkers of primarily diet or regular beverages according to their answers to these questions. Similar questions on caffeine content (caffeinated or decaffeinated) were asked for soft drinks, coffee, hot tea and iced-tea. Again, drinkers were classified accordingly. Finally, participants were asked what kind of sweetener they regularly add to coffee or tea. Possible answers were “didn't drink coffee or tea”, “didn't add any sweetener to coffee or tea”, “sugar or honey”, “Equal or aspartame”, “Saccharin or Sweet-n-Low”, and “other sweeteners”.

Coffee consumption was previously validated in this cohort among 1953 participants who also completed 24-h dietary recalls on two nonconsecutive days [23]. The Spearman correlation coefficients between these two methods were 0.80 for coffee-0.64 for caffeinated coffee, and 0.48 for decaffeinated coffee. Further, coffee drinking in this cohort has been linked to lower total mortality [24] and lower risk of Parkinson's disease [12] and certain types of cancer [25], [26].

In addition to dietary habits, the baseline questionnaire collected information on demographics, lifestyle, self-evaluated health status, and diagnosis of major chronic diseases. For smoking, participants were asked whether they had smoked more than 100 cigarettes during their lifetime. Ever smokers were asked about current smoking status and typical number of cigarettes per day. Former smokers were asked the year of cessation. Consumption of beer, wine and liquor was asked as part of the baseline food frequency questionnaire. For physical activity, the questionnaire inquired whether in the prior 12 months the subject participated in activities at work or home that lasted at least 20 minutes or increased breath, heart rate, or caused sweating. Body mass index (BMI) was calculated as weight in kilograms divided by height in squared meters.

### Statistical analysis

In the analyses, drinking frequency was categorized as (cans or cups/day): none (reference), <1, 1, 2–3, and ≥4. To reduce the possibility that early symptoms of depression might have affected drinking habit, we only included incident diagnoses since 2000, i.e. those diagnoses that were made at least four years after baseline. Multivariate odds ratios (OR) and 95% confidence intervals (CI) were derived from logistic regression models, adjusting for age at baseline (5-year groups), sex, race (non-Hispanic Whites vs. others), education level (<8 years, 8–11 years, 12 years or high school, post-high school or some college, college and post graduate), marital status (married or living as married, widowed, divorced, separated, or never married), smoking status (never smokers; past smokers with years since last smoking: ≥35, 30–34, 20–29, 10–19, 1–9; current smokers with the numbers of cigarettes/day: 1–10, 11–20, >20), consumption of beer, liquor and wine (drinks/day: none, <1, 1–1.9, 2–2.9, ≥3), physical activity (times/week: never/rarely, <1, 1–2, 3–4, and ≥5), and BMI (kg/m^2^: <25, 25–29.9, ≥30), and energy intake (quintiles). Further adjustment for self-evaluated health status (excellent, very good, good, fair or poor) and the presence of diabetes, heart disease, and cancer at baseline, had did not materially change the results. As these conditions might be in the pathway between drink consumptions and depression, we presented these analyses as supplemental materials (Tables S1–S4). The statistical significance for a monotonic trend was examined by including the mid-point of each exposure category as a continuous variable in the regression model.

In addition to the overall analysis, we conducted stratified analyses according to the specific subtype of each beverage that the participant drank more than half of the time: regular vs. diet, or caffeinated vs. decaffeinated. These analyses were pre-planned. Finally, we conducted post-hoc analyses to examine specific type of sweeteners regularly added to coffee or tea in relation to the risk of depression. All statistical analyses were performed by using the Statistical Analysis Systems (SAS) release 9.3 (SAS Institute, Cary, NC, USA) with two-tailed α of 0.05.

## Results

Table 1 shows baseline population characteristics. As expected, depression was more likely to occur in women, current smokers, participants with low education level, and those who divorced. Further, depression was associated with higher BMI, less physical activity, poorer general health status, and a history of diabetes, CVD, or cancer.

**Table 1:** Baseline population characteristics of the NIH-AARP Diet and Health Study, 1995–2006.

	Depression	
	Cases	Controls
N	11,311	252,612
Baseline age in years, mean (SD)	61.1 (5.6)	61.6 (5.3)
Men, %	48.5	61.2
Race, %		
Non-Hispanic Whites	92.9	92.6
Others	6.0	6.5
Missing	1.1	1.0
Education, %		
High school or less	25.3	21.7
Post high school	10.9	9.3
Some college	25.3	22.5
College and above	35.9	44.2
Missing	2.6	2.2
Marital status, %		
Married/living as married	67.7	71.9
Widowed	10.0	9.9
Divorced	17.1	12.9
Separated	4.7	4.8
Never married	0.0	0.0
Unknown	0.5	0.5
Smokers, %		
Never	33.7	38.8
Past	51.5	50.8
Current	13.6	9.1
Missing	1.2	1.2
Body mass index (kg/m^2^), Mean (SD)	27.5 (5.4)	26.9 (4.8)
Physical activity, %		
Never or rarely	20.5	14.8
1–3 times/month	15.4	13.3
1–2 times/week	21.5	22.1
3–4 times/week	25.5	28.4
≥5 times/week	16.3	20.7
Missing	0.8	0.7
Self-reported health status, %		
Excellent/very good	43.0	58.3
Good	39.3	32.6
Fair	14.7	7.2
Poor	1.6	0.6
Missing	1.4	1.2
History of diabetes, %	9.6	6.9
History of heart disease, %	13.8	11.6
History of cancer, %	7.9	7.6
Energy intake (kcal/day), Mean (SD)	1799 (931)	1755 (822)
Alcoholic beverages (drinks/day), %		
None	24.4	21.0
<1	55.1	54.4
1–1.99	10.4	12.9
2–2.99	3.5	4.0
≥3	6.6	7.7

Overall, higher consumption of soft or fruit drinks at baseline was monotonically associated with a higher risk of depression (Table 2). The multivariate OR between extreme drinking categories (≥4 cans/day vs. none) was 1.30 (1.17–1.44) for soft drinks, and 1.38 (1.15–1.65) for fruit drinks (both *P* for trend<0.0001). For soft drinks, the risk increase was statistically significant for all categories of >1 can per day. In contrast, coffee drinking was weakly associated with a lower risk for depression (OR for ≥4 cups/day vs. none  = 0.91 (0.84–0.98), *P* for trend<0.0001). Overall, drinking hot tea or iced tea was not associated with the risk of depression. Similar results were found in both genders with the exception of fruit drinks where the association was limited to men.

**Table 2:** Odds ratios^a^ and 95% confidence intervals of depression according to baseline beverage consumption in the NIH-AARP Diet and Health Study, 1995–2006.

	Overall			Men			Women					
Beverages	Case/control^b^	OR	95% CI	Case/Control	OR	95% CI	Case/Control		OR		95% CI	
Soft drinks (cans/day)												
None	985/23633	1.00		358/11123	1.00			627/12510		1.00		
<1	8024/188196	1.06	0.99–1.14	3849/116075	1.03	0.92–1.15		4175/72121		1.08		0.99–1.18
1	549/10060	1.26	1.13–1.40	300/6760	1.24	1.05–1.45		249/3300		1.26		1.08–1.47
2–3	881/16050	1.26	1.15–1.39	502/10991	1.27	1.11–1.47		379/5059		1.22		1.06–1.39
≥4	653/10524	1.30	1.17–1.44	378/7225	1.32	1.13–1.53		275/3299		1.25		1.07–1.45
*P* for trend	<0.0001			<0.0001				<0.0001				
Fruit drinks (cans/day)												
None	6567/144579	1.00		3035/84491	1.00			3532/60088		1.00		
<1	4181/97416	0.98	0.94–1.02	2144/63318	0.96	0.90–1.01		2037/34098		1.01		0.96–1.07
1	175/3341	1.11	0.95–1.30	105/2251	1.19	0.97–1.45		70/1090		1.00		0.78–1.28
2–3	62/1127	1.10	0.85–1.43	32/631	1.27	0.89–1.82		30/496		0.95		0.65–1.38
≥4	135/1907	1.38	1.15–1.65	89/1327	1.56	1.25–1.95		46/580		1.11		0.82–1.51
*P* for trend	<0.0001			<0.0001				0.67				
Hot tea (cups/day)												
None	7246/163165	1.00		3917/108660	1.00			3329/54505		1.00		
<1	2087/46465	1.01	0.96–1.06	837/24833	1.01	0.93–1.09		1250/21632		1.00		0.93–1.07
1	951/20948	1.01	0.94–1.08	355/10607	1.00	0.90–1.12		596/10341		1.01		0.92–1.10
2–3	761/17099	0.97	0.90–1.05	276/8150	1.00	0.89–1.14		485/8949		0.94		0.85–1.04
≥4	203/3697	1.14	0.98–1.31	73/1693	1.21	0.95–1.53		130/2004		1.10		0.92–1.32
*P* for trend	0.57			0.32				0.89				
Iced tea (cups/day)												
None	4299/98282	1.00		2110/59885	1.00			2189/38397		1.00		
<1	3377/76916	1.01	0.96–1.06	1649/48699	0.97	0.91–1.04		1728/28217		1.05		0.98–1.12
1	985/22750	0.96	0.89–1.03	467/13060	1.01	0.91–1.12		518/9690		0.92		0.84–1.02
2–3	1910/40669	0.98	0.93–1.04	910/24298	0.99	0.91–1.07		1000/16371		0.99		0.91–1.07
≥4	701/13226	1.03	0.95–1.12	337/8331	0.96	0.86–1.09		364/4895		1.10		0.98–1.23
*P* for trend	0.96			0.69				0.59				
Coffee (cups/day)												
None	1178/25783	1.00		481/14048	1.00			697/11735		1.00		
<1	1898/40626	1.07	0.99–1.15	852/23863	1.07	0.95–1.20		1046/16763		1.06		0.96–1.18
1	1895/41087	1.03	0.95–1.11	840/23284	1.06	0.94–1.19		1055/17803		1.00		0.91–1.11
2–3	4504/105351	0.93	0.87–1.00	2294/66195	0.97	0.87–1.08		2210/39156		0.90		0.82–0.99
≥4	1793/38992	0.91	0.84–0.98	1003/26901	0.90	0.80–1.01		790/12091		0.93		0.84–1.04
*P* for trend	<0.0001			0.0002				0.0006				

Abbreviations: CI, confidence interval; OR, odds ratio;

a Adjusted for age at baseline, sex, race, education, marital status, smoking, alcoholic beverage intake, physical activity, body mass index, and energy intake.

b Numbers may not add up to total due to missing.

For fruit drinks and sweetened iced tea, further analyses suggested that the association was limited to individuals who drank primarily diet beverages (Table 3). The ORs comparing the ≥4 cans/cups per day versus none were 1.51 (1.18–1.92) for diet versus 1.08 (0.79–1.46) for regular fruit drinks, and 1.25 (1.10–1.41) for diet versus 0.94 (0.83–1.08) for regular sweetened iced-tea. This differential pattern was less evident for soft drinks where the corresponding ORs were 1.31 (1.16–1.47) for diet and 1.22 (1.03–1.45) for regular soft drinks.

**Table 3:** Odds ratios^a^ and 95% confidence intervals of depression according to baseline consumption of regular or diet sweetened beverages in the NIH-AARP Diet and Health Study, 1995–2006.

	Regular drinks				Diet drinks		
Beverage	Case/Control^b^	OR	95% CI		Case/Control	OR	95% CI
Soft drinks (cans/day)							
None	985/23633	1.00			985/23633	1.00	
<1	3188/82825	0.97	0.89–1.04		4434/95590	1.14	1.06–1.23
1	189/3442	1.23	1.04–1.45		355/6500	1.26	1.11–1.43
2–3	230/4335	1.16	1.00–1.36		643/11575	1.29	1.16–1.43
≥4	181/2829	1.22	1.03–1.45		465/7578	1.31	1.16–1.47
*P* for trend		0.0001				<0.0001	
Fruit drinks (cans/day)							
None	6567/144580	1.00			6567/144579	1.00	
<1	2018/50147	0.92	0.87–0.96		1225/25598	1.06	0.99–1.13
1	87/1899	0.96	0.77–1.19		54/1034	1.09	0.83–1.44
2–3	4/124	0.58	0.21–1.57		56/957	1.18	0.90–1.55
≥4	45/773	1.08	0.79–1.46		73/958	1.51	1.18–1.92
*P* for trend		0.84				0.0005	
Sweetened iced tea (cups/day)							
None	4299/98282	1.00			4299/98282	1.00	
<1	1167/28082	0.96	0.90–1.03		1308/25980	1.14	1.07–1.22
1	378/8592	0.97	0.87–1.09		373/7933	1.04	0.93–1.16
2–3	689/15995	0.90	0.83–0.98		770/14385	1.12	1.04–1.22
≥4	254/5214	0.94	0.83–1.08		291/4521	1.25	1.10–1.41
*P* for trend		0.036				<0.0001	

Abbreviations: CI, confidence interval; OR, odds ratio.

a Adjusted for age at baseline, sex, race, education, marital status, smoking, alcoholic beverage intake, physical activity and body mass index, and energy intake.

b Numbers may not add up to total due to missing.

In the analyses according to caffeine content, frequent drinkers of both types of coffee (primarily either caffeinated or decaffeinated) had a slightly lower depression risk than nondrinkers (Table 4). In contrast, consumption of both caffeinated and decaffeinated soft drinks was related to higher risk of depression. Interestingly, drinking either iced-tea or hot tea was monotonically associated with higher risk of depression among drinkers who primarily drank decaffeinated teas. In contrast, among participants who drank primarily caffeinated teas, we found a null association with hot tea and a weak inverse association with iced tea.

**Table 4:** Odds ratios^a^ and 95% confidence intervals of depression according to baseline consumption of caffeinated or decaffeinated beverages in the NIH-AARP Diet and Health Study, 1995–2006.

	Caffeinated drinks				Decaffeinated drinks		
Beverages	Case/Control^b^	OR	95% CI		Case/Control	OR	95% CI
Coffee (cups/day)							
None	1178/25783	1.00			1178/25783	1.00	
<1	770/16789	1.04	0.95–1.14		1006/21047	1.09	1.00–1.19
1	1072/24418	0.98	0.89–1.06		789/15877	1.10	1.00–1.21
2–3	3049/72917	0.91	0.84–0.98		1371/30352	0.98	0.90–1.07
≥4	1380/29461	0.90	0.83–0.98		372/8736	0.88	0.78–1.00
*P* for trend		0.0026				0.0029	
Soft drinks (cans/day)							
None	985/23633	1.00			985/23633	1.00	
<1	3929/96355	1.02	0.94–1.10		3531/78095	1.12	1.04–1.20
1	276/5098	1.23	1.07–1.42		252/4632	1.26	1.09–1.46
2–3	420/8408	1.13	1.00–1.27		429/7182	1.38	1.23–1.56
≥4	350/5533	1.28	1.12–1.46		279/4633	1.29	1.12–1.49
*P* for trend			<0.0001			<0.0001	
Iced tea (cups/day)							
None	4299/98282	1.00			4299/98282	1.00	
<1	2529/59555	0.98	0.93–1.03		763/15620	1.12	1.03–1.21
1	697/16557	0.94	0.86–1.02		276/5718	1.07	0.94–1.21
2–3	1295/29093	0.93	0.87–0.99		593/10905	1.15	1.05–1.26
≥4	445/9406	0.91	0.82–1.00		247/3569	1.37	1.20–1.57
*P* for trend		0.0089				<0.0001	
Hot tea (cups/day)							
None	7246/163165	1.00			7246/163165	1.00	
<1	1216/28851	0.95	0.90–1.02		811/16413	1.09	1.01–1.18
1	523/13075	0.90	0.82–0.99		400/7372	1.18	1.06–1.31
2–3	450/11115	0.89	0.81–0.98		289/5500	1.12	0.99–1.26
≥4	138/2596	1.10	0.92–1.31		61/985	1.27	0.98–1.65
*P* for trend		0.14				0.0006	

Abbreviations: CI, confidence interval; OR, odds ratio.

a Adjusted for age at baseline, sex, race, education, marital status, smoking, alcoholic beverage intake, physical activity, body mass index, and energy intake.

b Numbers may not add up to total due to missing.

Similar results were observed when we examined which types of sweeteners were regularly added to coffee or tea (Table 5). Compared to those who did not drink coffee or tea, drinkers who did not add any sweetener had a lower risk for depression, whereas those who regularly added artificial sweeteners had higher risks. Adding sugar or honey was not related to the risk of depression.

**Table 5:** Odds ratios^a^ and 95% confidence intervals of depression according to types of sweetener added to coffee or tea in the NIH-AARP Diet and Health Study, 1995–2006.

	Overall			Men			Women		
Sweeteners	Case/control	OR	95% CI	Case/Control	OR	95% CI	Case/Control	OR	95% CI
Non-drinkers	408/9297	1.00		219/6044	1.00		189/3253	1.00	
None	4580/118082	0.86	0.77–0.95	2179/71644	0.84	0.73–0.97	2401/46438	0.88	0.75–1.03
Sugar or honey	2603/59917	0.93	0.83–1.04	1390/39768	0.89	0.77–1.03	1213/20149	0.98	0.83–1.14
Equal or aspartame	1778/30199	1.24	1.11–1.39	805/16989	1.27	1.09–1.48	973/13210	1.22	1.03–1.43
Saccharin or Sweet-n-Low	1751/31624	1.14	1.02–1.27	825/18430	1.17	1.01–1.37	926/13194	1.11	0.94–1.31
Other sweeteners	145/2349	1.23	1.01–1.49	51/1216	1.07	0.78–1.46	94/1133	1.35	1.04–1.74

Abbreviations: CI, confidence interval; OR, odds ratio;

a Adjusted for age at baseline, sex, race, education, marital status, smoking, alcoholic beverage intake, physical activity, body mass index, and energy intake.

In all analyses, further adjusting for self-reported health status and the presence of diabetes, heart disease, and cancer only modestly attenuated the association (Tables S1–S4).

## Discussion

This large study included a total of 11,311 incident depression cases identified from self-reports. Although we did not conduct diagnostic confirmation, depression in this population was associated with female gender, lower education, smoking, lack of physical activity, obesity, the presence of major chronic diseases and poor health status. These data indirectly support the validity of case identification in this cohort.

Major strengths of this study include the very large sample size, prospective data collection, and detailed analyses. In comparison to retrospective data collection, prospective exposure assessment is less prone to recall bias and reverse causation. Moreover, we only included depression diagnoses occurring at least four years after exposure assessment which further reduced the potential influence of reverse causation on our analyses.

Only a few cross-sectional analyses have published data on soft drinks and depression like outcomes. A study in Australia reported that adults who consumed over a liter of soft drink per day had approximately 60% higher prevalence of depression, suicidal ideation, or mental problems [14]. One study in China and one in Norway reported a J-shaped association among adolescents with a slightly higher prevalence of suicidal plan or act [15] or mental problems [16] among never or rare drinkers of soft drinks, but a much higher risk among heavy drinkers.

To the best of our knowledge, this is the first prospective study to observe a modest positive association between frequent drinking of sweetened beverages and depression. In general, further analyses suggest that the observation may be more relevant to diet drinks. Unlike sugar-sweetened drinks, diet drinks often use artificial sweeteners such as aspartame and saccharin for the sweet taste and are calorie-free. Our further analysis found that adding these artificial sweeteners to coffee or tea, but not adding sugar or honey, was associated with higher risk of depression. Various effects of artificial sweeteners, including neurological effects, have been suspected [6], [7]. For example, aspartame may modulate brain neurotransmitters such as dopamine and serotonin, although data have been controversial and inconsistent [27].

There are also possible alternative explanations. One possibility is that depressed individuals may crave sweet beverages, and one may speculate that this may occur even years before receiving a diagnosis of depression. We cannot exclude this possibility of reverse causation despite the fact that the analyses only included cases diagnosed after 2000. However, this explanation cannot explain some differential results on diet versus regular sweetened beverages, or on adding artificial sweeteners as compared to adding sugar or honey to tea or coffee. Consumption of sweetened beverages is associated with a variety of socio-economic and lifestyle factors and may contribute to obesity, diabetes and poor health, which in turn could contribute to the development of depression. Although we cannot exclude the possibility of residual confounding, we adjusted for these factors in the analyses.

Several studies have examined coffee or tea consumption in relation to depression with inconsistent results. Most were cross-sectional, which further complicates interpretation of the data [28], [29], [30], [31], [32], [33], [34], [35]. Three prospective studies have been published. In a study of Finnish men (49 cases), heavy coffee drinkers had approximately 70% lower risk for depression than nondrinkers [21]. In the Nurses' Health Study (2607 cases): women who drank >4 cups coffee per day had a 20% lower risk of depression than those who drank never or rarely coffee [20]. The associations were observed for caffeinated but not decaffeinated coffee, although fewer participants drank decaffeinated coffee. Caffeinated tea, soft drinks, or caffeine from non-coffee sources were not related to depression in the study [20]. The third study (363 cases) reported that tea drinking was associated with less depression among female breast cancer survivors in China [36].

Compared to previous studies, our study is much larger and included both men and women. In the analyses, coffee drinking was linked to a slightly lower risk of depression. A similar weak association was also observed with consumption of caffeinated iced-tea, whereas the association with decaffeinated iced-tea was in the opposite direction. Coffee contains large amounts of caffeine which is a well-known brain stimulant. Caffeine and its major metabolites act on adenosine receptors in the brain [37] and increase plasticity of hippocampal CA2 neurons [38], which may in turn contribute to lower risk of depression among coffee drinkers. In addition to caffeine, coffee and tea contain many antioxidants and phytochemicals which may also be responsible for our observations.

In addition to self-reported outcome, there are several other limitations. We asked only about beverage consumption in the year prior to baseline and therefore we did not capture consumption history or changes in drinking habits over time. Measurement error was thus unavoidable. This may be particularly true for assessing diet versus regular beverages or of caffeinated versus decaffeinated drinks because we classified participants according to which particular subtype they drank more than half of the time. Therefore results on subtypes of drinks must be interpreted within context. Nevertheless, given the prospective design, these measurement errors are likely to be random and therefore would tend to attenuate true associations. The present analysis was conducted among participants of the cohort's follow-up survey and therefore selection bias was possible, the direction of which could not be readily predicted. Finally, the study included AARP members from selected regions of the US, therefore the generalizability of these results, particularly to younger populations, need further investigation.

In conclusion, in this large study of U.S. older adults, frequent consumption of sweetened beverages was associated with a modestly higher risk of depression, and coffee consumption with a slightly lower risk. As these drinks are commonly consumed, confirmation and further investigations are warranted.

## Supporting Information

Table S1 **Odds ratios and 95% confidence intervals of depression according to baseline beverage consumption, further adjusted for self-reported health status, diabetes, heart disease, and cancer.**
(DOCX)

Table S2 **Odds ratios and 95% confidence intervals of depression according to baseline consumption of regular or diet sweetened beverages, further adjusted for self-reported health status, diabetes, heart disease, and cancer.**
(DOCX)

Table S3 **Odds ratios and 95% confidence intervals of depression according to baseline consumption of caffeinated or decaffeinated beverages, further adjusted for self-reported health status, diabetes, heart disease, and cancer.**
(DOCX)

Table S4 **Odds ratios and 95% confidence intervals of depression according to types of sweetener added to coffee or tea, further adjusted for self-reported health status, diabetes, heart disease, and cancer.**
(DOCX)

## References

[1] Popkin BM, Armstrong LE, Bray GM, Caballero B, Frei B, et al (2006) A new proposed guidance system for beverage consumption in the United States. The American journal of clinical nutrition 83: 529–542. 16522898 10.1093/ajcn.83.3.529

[2] Malik VS, Popkin BM, Bray GA, Despres JP, Willett WC, et al (2010) Sugar-sweetened beverages and risk of metabolic syndrome and type 2 diabetes: a meta-analysis. Diabetes care 33: 2477–2483. 20693348 10.2337/dc10-1079 PMC2963518

[3] Malik VS, Schulze MB, Hu FB (2006) Intake of sugar-sweetened beverages and weight gain: a systematic review. The American journal of clinical nutrition 84: 274–288. 16895873 10.1093/ajcn/84.1.274 PMC3210834

[4] Mozaffarian D, Hao T, Rimm EB, Willett WC, Hu FB (2011) Changes in diet and lifestyle and long-term weight gain in women and men. The New England journal of medicine 364: 2392–2404. 21696306 10.1056/NEJMoa1014296 PMC3151731

[5] Qi Q, Chu AY, Kang JH, Jensen MK, Curhan GC, et al. (2012) Sugar-sweetened beverages and genetic risk of obesity. N Engl J Med 367: \1387–1396. 10.1056/NEJMoa1203039 PMC3518794 22998338

[6] Whitehouse CR, Boullata J, McCauley LA (2008) The potential toxicity of artificial sweeteners. AAOHN J 56: 251–259. 18604921 10.3928/08910162-20080601-02

[7] Humphries P, Pretorius E, Naude H (2008) Direct and indirect cellular effects of aspartame on the brain. European journal of clinical nutrition 62: 451–462. 17684524 10.1038/sj.ejcn.1602866

[8] Huxley R, Lee CM, Barzi F, Timmermeister L, Czernichow S, et al (2009) Coffee, decaffeinated coffee, and tea consumption in relation to incident type 2 diabetes mellitus: a systematic review with meta-analysis. Archives of internal medicine 169: 2053–2063. 20008687 10.1001/archinternmed.2009.439

[9] Lara DR (2010) Caffeine, mental health, and psychiatric disorders. Journal of Alzheimer's disease: JAD 20 Suppl 1S239–248. 10.3233/JAD-2010-1378 20164571

[10] Ascherio A, Zhang SM, Hernán MA, Kawachi I, Colditz GA, et al (2001) Prospective study of caffeine consumption and risk of Parkinson's disease in men and women. Ann Neurol 50: 56–63. 11456310 10.1002/ana.1052

[11] Ross GW, Abbott RD, Petrovitch H, Morens DM, Grandinetti A, et al (2000) Association of coffee and caffeine intake with the risk of Parkinson disease. JAMA 283: 2674–2679. 10819950 10.1001/jama.283.20.2674

[12] Liu R, Guo X, Park Y, Huang X, Sinha R, et al (2012) Caffeine intake, smoking, and risk of Parkinson disease in men and women. American journal of epidemiology 175: 1200–1207. 22505763 10.1093/aje/kwr451 PMC3370885

[13] Santos C, Costa J, Santos J, Vaz-Carneiro A, Lunet N (2010) Caffeine intake and dementia: systematic review and meta-analysis. Journal of Alzheimer's disease: JAD 20 Suppl 1S187–204. 20182026 10.3233/JAD-2010-091387

[14] Shi Z, Taylor AW, Wittert G, Goldney R, Gill TK (2010) Soft drink consumption and mental health problems among adults in Australia. Public health nutrition 13: 1073–1079. 20074392 10.1017/S1368980009993132

[15] Pan X, Zhang C, Shi Z (2011) Soft drink and sweet food consumption and suicidal behaviours among Chinese adolescents. Acta paediatrica 100: e215–222. 21627691 10.1111/j.1651-2227.2011.02369.x

[16] Lien L, Lien N, Heyerdahl S, Thoresen M, Bjertness E (2006) Consumption of soft drinks and hyperactivity, mental distress, and conduct problems among adolescents in Oslo, Norway. American journal of public health 96: 1815–1820. 17008578 10.2105/AJPH.2004.059477 PMC1586153

[17] Klatsky AL, Armstrong MA, Friedman GD (1993) Coffee, tea, and mortality. Annals of epidemiology 3: 375–381. 8275213 10.1016/1047-2797(93)90064-b

[18] Kawachi I, Willett WC, Colditz GA, Stampfer MJ, Speizer FE (1996) A prospective study of coffee drinking and suicide in women. Archives of internal medicine 156: 521–525. 8604958

[19] Tanskanen A, Tuomilehto J, Viinamaki H, Vartiainen E, Lehtonen J, et al (2000) Heavy coffee drinking and the risk of suicide. European journal of epidemiology 16: 789–791. 11297219 10.1023/a:1007614714579

[20] Lucas M, Mirzaei F, Pan A, Okereke OI, Willett WC, et al (2011) Coffee, caffeine, and risk of depression among women. Arch Intern Med 171: 1571–1578. 21949167 10.1001/archinternmed.2011.393 PMC3296361

[21] Ruusunen A, Lehto SM, Tolmunen T, Mursu J, Kaplan GA, et al (2010) Coffee, tea and caffeine intake and the risk of severe depression in middle-aged Finnish men: the Kuopio Ischaemic Heart Disease Risk Factor Study. Public health nutrition 13: 1215–1220. 20359377 10.1017/S1368980010000509

[22] Schatzkin A, Subar AF, Thompson FE, Harlan LC, Tangrea J, et al (2001) Design and serendipity in establishing a large cohort with wide dietary intake distributions: the National Institutes of Health-American Association of Retired Persons Diet and Health Study. Am J Epidemiol 154: 1119–1125. 11744517 10.1093/aje/154.12.1119

[23] Thompson FE, Kipnis V, Midthune D, Freedman LS, Carroll RJ, et al (2008) Performance of a food-frequency questionnaire in the US NIH-AARP (National Institutes of Health-American Association of Retired Persons) Diet and Health Study. Public Health Nutr 11: 183–195. 17610761 10.1017/S1368980007000419

[24] Freedman ND, Park Y, Abnet CC, Hollenbeck AR, Sinha R (2012) Association of coffee drinking with total and cause-specific mortality. The New England journal of medicine 366: 1891–1904. 22591295 10.1056/NEJMoa1112010 PMC3439152

[25] Sinha R, Cross AJ, Daniel CR, Graubard BI, Wu JW, et al (2012) Caffeinated and decaffeinated coffee and tea intakes and risk of colorectal cancer in a large prospective study. The American journal of clinical nutrition 96: 374–381. 22695871 10.3945/ajcn.111.031328 PMC3396445

[26] Gunter MJ, Schaub JA, Xue X, Freedman ND, Gaudet MM, et al (2012) A prospective investigation of coffee drinking and endometrial cancer incidence. International journal of cancer Journal international du cancer 131: E530–536. 22021096 10.1002/ijc.26482 PMC3288610

[27] Magnuson BA, Burdock GA, Doull J, Kroes RM, Marsh GM, et al (2007) Aspartame: a safety evaluation based on current use levels, regulations, and toxicological and epidemiological studies. Crit Rev Toxicol 37: 629–727. 17828671 10.1080/10408440701516184

[28] Benko CR, Farias AC, Farias LG, Pereira EF, Louzada FM, et al (2011) Potential link between caffeine consumption and pediatric depression: A case-control study. BMC pediatrics 11: 73. 21867528 10.1186/1471-2431-11-73 PMC3180267

[29] Greden JF, Fontaine P, Lubetsky M, Chamberlin K (1978) Anxiety and depression associated with caffeinism among psychiatric inpatients. The American journal of psychiatry 135: 963–966. 665843 10.1176/ajp.135.8.963

[30] Smith AP (2009) Caffeine, cognitive failures and health in a non-working community sample. Human psychopharmacology 24: 29–34. 19016251 10.1002/hup.991

[31] Whalen DJ, Silk JS, Semel M, Forbes EE, Ryan ND, et al (2008) Caffeine consumption, sleep, and affect in the natural environments of depressed youth and healthy controls. Journal of pediatric psychology 33: 358–367. 17947257 10.1093/jpepsy/jsmo86 PMC2492889

[32] Leibenluft E, Fiero PL, Bartko JJ, Moul DE, Rosenthal NE (1993) Depressive symptoms and the self-reported use of alcohol, caffeine, and carbohydrates in normal volunteers and four groups of psychiatric outpatients. The American journal of psychiatry 150: 294–301. 8422081 10.1176/ajp.150.2.294

[33] Luebbe AM, Bell DJ (2009) Mountain Dew or mountain don't? a pilot investigation of caffeine use parameters and relations to depression and anxiety symptoms in 5th- and 10th-grade students. The Journal of school health 79: 380–387. 19630872 10.1111/j.1746-1561.2009.00424.x

[34] Niu K, Hozawa A, Kuriyama S, Ebihara S, Guo H, et al (2009) Green tea consumption is associated with depressive symptoms in the elderly. The American journal of clinical nutrition 90: 1615–1622. 19828710 10.3945/ajcn.2009.28216

[35] Hintikka J, Tolmunen T, Honkalampi K, Haatainen K, Koivumaa-Honkanen H, et al (2005) Daily tea drinking is associated with a low level of depressive symptoms in the Finnish general population. European journal of epidemiology 20: 359–363. 15971509 10.1007/s10654-005-0148-2

[36] Chen X, Lu W, Zheng Y, Gu K, Chen Z, et al (2010) Exercise, tea consumption, and depression among breast cancer survivors. Journal of clinical oncology: official journal of the American Society of Clinical Oncology 28: 991–998. 20048185 10.1200/JCO.2009.23.0565 PMC2834436

[37] Morelli M, Carta AR, Kachroo A, Schwarzschild MA (2010) Pathophysiological roles for purines: adenosine, caffeine and urate. Progress in brain research 183: 183–208. 20696321 10.1016/S0079-6123(10)83010-9 PMC3102301

[38] Simons SB, Caruana DA, Zhao M, Dudek SM (2012) Caffeine-induced synaptic potentiation in hippocampal CA2 neurons. Nature neuroscience 15: 23–25. 10.1038/nn.2962 PMC3245784 22101644

