# Supplementary material for: Sweetened Beverages, Coffee, and Tea and Depression Risk among Older US Adults
Source: PLoS One. 2014 Apr 17;9(4):e94715. doi: 10.1371/journal.pone.0094715 (PMC3990543; doi:10.1371/journal.pone.0094715)
Supplement: Table S1 — Odds ratios and 95% confidence intervals of depression according to baseline beverage consumption, further adjusted for self-reported health status, diabetes, heart disease, and cancer. (DOCX) [file pone.0094715.s001.docx]

**Table S1. Odds ratios**^a^ **and 95% confidence intervals of depression according to baseline beverage consumption, further adjusted for self-reported health status, diabetes, heart disease, and cancer**

|  | Overall | | | |  | Men | | | | | |  | | Women | | | | | |  |  |  |  |  |  |  |  |
| --- | --- | --- | --- | --- | --- | --- | --- | --- | --- | --- | --- | --- | --- | --- | --- | --- | --- | --- | --- | --- | --- | --- | --- | --- | --- | --- | --- |
| Beverages | Case/control ^b^ | OR | | 95% CI |  | | Case /Control | | OR | | 95% CI | |  | | Case /Control | | OR | | 95% CI | |  |  |  |  |  |  |  |
| Soft drinks (cans/day) | | | | | | | | | | | | | | | | | | | |  |  |  |  |  |  |  |  |
| None | 985/23633 | 1.00 | |  |  | 358/11123 | | 1.00 | |  | |  | | 627/12510 | | 1.00 | |  | |  |  |  |  |  |  |  |  |
| < 1 | 8024/188196 | 1.04 | | 0.97-1.11 |  | 3849/116075 | | 1.00 | | 0.89-1.12 | |  | | 4175/72121 | | 1.06 | | 0.97-1.16 | |  |  |  |  |  |  |  |  |
| 1 | 549/10060 | 1.20 | | 1.08-1.34 |  | 300/6760 | | 1.18 | | 1.00-1.38 | |  | | 249/3300 | | 1.20 | | 1.03-1.40 | |  |  |  |  |  |  |  |  |
| 2-3 | 881/16050 | 1.20 | | 1.09-1.32 |  | 502/10991 | | 1.19 | | 1.03-1.37 | |  | | 379/5059 | | 1.18 | | 1.03-1.35 | |  |  |  |  |  |  |  |  |
| ≥ 4 | 653/10524 | 1.22 | | 1.10-1.36 |  | 378/7225 | | 1.22 | | 1.05-1.43 | |  | | 275/3299 | | 1.18 | | 1.02-1.38 | |  |  |  |  |  |  |  |  |
| *P* for trend | <0.0001 | | | |  | <0.0001 | | | | | |  | | 0.007 | | | | | |  |  |  |  |  |  |  |  |
| Fruit drinks (cans/day) | | | | | | | | | | | | | | | | | | | | | |  |  |  |  |  |  |
| None | 6567/144579 | 1.00 |  | |  | 3035/84491 | | 1.00 | |  | |  | | 3532/60088 | | 1.00 | | | |  | | |  |  |  |  |  |
| < 1 | 4181/97416 | 0.97 | 0.93-1.01 | |  | 2144/63318 | | 0.95 | | 0.90-1.01 | |  | | 2037/34098 | | 1.00 | | 0.94-1.06 | |  |  |  |  |  |  |  |  |
| 1 | 175/3341 | 1.07 | 0.91-1.25 | |  | 105/2251 | | 1.16 | | 0.95-1.42 | |  | | 70/1090 | | 0.96 | | 0.75-1.23 | |  |  |  |  |  |  |  |  |
| 2-3 | 62/1127 | 1.04 | 0.80-1.35 | |  | 32/631 | | 1.17 | | 0.82-1.68 | |  | | 30/496 | | 0.91 | | 0.63-1.33 | |  |  |  |  |  |  |  |  |
| ≥ 4 | 135/1907 | 1.31 | 1.10-1.57 | |  | 89/1327 | | 1.48 | | 1.19-1.85 | |  | | 46/580 | | 1.07 | | 0.78-1.45 | |  |  |  |  |  |  |  |  |
| *P* for trend | 0.002 | | | |  | 0.0001 | | | | | |  | | 0.96 | | | | | |  | | |  |  |  |  |  |
| Hot tea (cups/day) | | | | | | | | | | | | | | | | | | | |  |  |  |  |  |  |  |  |
| None | 7246/163165 | 1.00 | |  |  | 3917/108660 | | 1.00 | |  | |  | | 3329/54505 | | 1.00 | |  | |  |  |  |  |  |  |  |  |
| < 1 | 2087/46465 | 0.99 | | 0.94-1.04 |  | 837/24833 | | 0.99 | | 0.92-1.07 | |  | | 1250/21632 | | 0.98 | | 0.92-1.05 | |  |  |  |  |  |  |  |  |
| 1 | 951/20948 | 0.99 | | 0.92-1.06 |  | 355/10607 | | 0.98 | | 0.88-1.10 | |  | | 596/10341 | | 0.99 | | 0.90-1.08 | |  |  |  |  |  |  |  |  |
| 2-3 | 761/17099 | 0.95 | | 0.88-1.03 |  | 276/8150 | | 0.99 | | 0.87-1.12 | |  | | 485/8949 | | 0.93 | | 0.84-1.03 | |  |  |  |  |  |  |  |  |
| ≥ 4 | 203/3697 | 1.14 | | 0.98-1.31 |  | 73/1693 | | 1.20 | | 0.94-1.52 | |  | | 130/2004 | | 1.11 | | 0.92-1.33 | |  |  |  |  |  |  |  |  |
| *P* for trend | 0.85 | | | |  | 0.50 | | | | | |  | | 0.75 | | | | | |  |  |  |  |  |  |  |  |
| Iced tea (cups/day) | | | | | | | | | | | | | | | | | | | | | |  |  |  |  |  |  |
| None | 4299/98282 | 1.00 |  | |  | 2110/59885 | | 1.00 | |  | |  | | 2189/38397 | | 1.00 | | | |  | | |  |  |  |  |  |
| < 1 | 3377/76916 | 1.00 | 0.96-1.05 | |  | 1649/48699 | | 0.96 | | 0.90-1.03 | |  | | 1728/28217 | | 1.04 | | 0.98-1.11 | |  |  |  |  |  |  |  |  |
| 1 | 985/22750 | 0.95 | 0.89-1.03 | |  | 467/13060 | | 1.00 | | 0.90-1.11 | |  | | 518/9690 | | 0.92 | | 0.83-1.01 | |  |  |  |  |  |  |  |  |
| 2-3 | 1910/40669 | 0.98 | 0.92-1.03 | |  | 910/24298 | | 0.97 | | 0.90-1.06 | |  | | 1000/16371 | | 0.98 | | 0.91-1.06 | |  |  |  |  |  |  |  |  |
| ≥ 4 | 701/13226 | 1.02 | 0.93-1.10 | |  | 337/8331 | | 0.95 | | 0.84-1.07 | |  | | 364/4895 | | 1.09 | | 0.97-1.23 | |  |  |  |  |  |  |  |  |
| *P* for trend | 0.79 | | | |  | 0.45 | | | | | |  | | 0.66 | | | | | |  |  |  |  |  |  |  |  |
| Coffee (cups/day) | | | | | | | | | | | | | | | | | | | |  |  |  |  |  |  |  |  |
| None | 1178/25783 | 1.00 | |  |  | 481/14048 | | 1.00 | |  | |  | | 697/11735 | | 1.00 | |  | |  |  |  |  |  |  |  |  |
| < 1 | 1898/40626 | 1.04 | | 0.97-1.13 |  | 852/23863 | | 1.04 | | 0.92-1.16 | |  | | 1046/16763 | | 1.05 | | 0.95-1.16 | |  |  |  |  |  |  |  |  |
| 1 | 1895/41087 | 1.01 | | 0.94-1.09 |  | 840/23284 | | 1.03 | | 0.92-1.16 | |  | | 1055/17803 | | 1.00 | | 0.90-1.10 | |  |  |  |  |  |  |  |  |
| 2-3 | 4504/105351 | 0.94 | | 0.88-1.01 |  | 2294/66195 | | 0.97 | | 0.88-1.08 | |  | | 2210/39156 | | 0.92 | | 0.84-1.01 | |  |  |  |  |  |  |  |  |
| ≥ 4 | 1793/38992 | 0.94 | | 0.87-1.01 |  | 1003/26901 | | 0.92 | | 0.82-1.04 | |  | | 790/12091 | | 0.97 | | 0.87-1.08 | |  |  |  |  |  |  |  |  |
| *P* for trend | 0.0004 | | | |  | 0.01 | | | | | |  | | 0.02 | | | | | |  |  |  |  |  |  |  |  |

Abbreviations: CI, confidence interval; OR, odds ratio.

^a^ Adjusted for age at baseline, sex, race, education, marital status, smoking, alcoholic beverage intake, physical activity, body mass index, energy intake, self-reported health status, diabetes, heart disease, and cancer.

^b^ Numbers may not add up to total due to missing
