# Supplementary material for: Sweetened Beverages, Coffee, and Tea and Depression Risk among Older US Adults
Source: PLoS One. 2014 Apr 17;9(4):e94715. doi: 10.1371/journal.pone.0094715 (PMC3990543; doi:10.1371/journal.pone.0094715)
Supplement: Table S2 — Odds ratios and 95% confidence intervals of depression according to baseline consumption of regular or diet sweetened beverages, further adjusted for self-reported health status, diabetes, heart disease, and cancer. (DOCX) [file pone.0094715.s002.docx]

**Table S2. Odds ratios** ^a^ **and 95% confidence intervals of depression according to baseline consumption of regular or diet sweetened beverages, further adjusted for self-reported health status, diabetes, heart disease, and cancer**

|  | Regular drinks | | |  | Diet drinks | | |
| --- | --- | --- | --- | --- | --- | --- | --- |
| Beverage | Case/Control ^b^ | OR | 95% CI |  | Case /Control | OR | 95% CI |
| Soft drinks (cans/day) | | | | | | | |
| None | 985/23633 | 1.00 |  |  | 985/23633 | 1.00 |  |
| < 1 | 3188/82825 | 0.95 | 0.88-1.03 |  | 4434/95590 | 1.11 | 1.03-1.19 |
| 1 | 189/3442 | 1.18 | 1.00-1.40 |  | 355/6500 | 1.21 | 1.06-1.37 |
| 2-3 | 230/4335 | 1.12 | 0.96-1.31 |  | 643/11575 | 1.22 | 1.10-1.36 |
| ≥ 4 | 181/2829 | 1.17 | 0.98-1.39 |  | 465/7578 | 1.24 | 1.10-1.39 |
| *P* for trend |  | 0.001 |  |  |  | 0.0004 |  |
| Fruit drinks (cans/day) | | | | | | | |
| None | 6567/144580 | 1.00 |  |  | 6567/144579 | 1.00 |  |
| < 1 | 2018/50147 | 0.92 | 0.87-0.97 |  | 1225/25598 | 1.03 | 0.97-1.10 |
| 1 | 87/1899 | 0.95 | 0.76-1.18 |  | 54/1034 | 1.04 | 0.79-1.37 |
| 2-3 | 4/124 | 0.58 | 0.21-1.58 |  | 56/957 | 1.11 | 0.84-1.45 |
| ≥ 4 | 45/773 | 1.07 | 0.78-1.45 |  | 73/958 | 1.40 | 1.10-1.79 |
| *P* for trend |  | 0.90 |  |  |  | 0.008 |  |
| Sweetened iced tea (cups/day) | | |  |  |  |  |  |
| None | 4299/98282 | 1.00 |  |  | 4299/98282 | 1.00 |  |
| < 1 | 1167/28082 | 0.96 | 0.90-1.03 |  | 1308/25980 | 1.12 | 1.05-1.20 |
| 1 | 378/8592 | 0.99 | 0.88-1.10 |  | 373/7933 | 1.02 | 0.91-1.14 |
| 2-3 | 689/15995 | 0.91 | 0.83-0.99 |  | 770/14385 | 1.10 | 1.02-1.19 |
| ≥ 4 | 254/5214 | 0.96 | 0.84-1.09 |  | 291/4521 | 1.22 | 1.08-1.38 |
| *P* for trend |  | 0.07 |  |  |  | 0.0004 |  |

Abbreviations: CI, confidence interval; OR, odds ratio

^a^ Adjusted for age at baseline, sex, race, education, marital status, smoking, alcoholic beverage intake, physical activity and body mass index, energy intake, self-reported health status, diabetes, heart disease, and cancer.

^b^ Numbers may not add up to total due to missing.
