# Supplementary material for: Sweetened Beverages, Coffee, and Tea and Depression Risk among Older US Adults
Source: PLoS One. 2014 Apr 17;9(4):e94715. doi: 10.1371/journal.pone.0094715 (PMC3990543; doi:10.1371/journal.pone.0094715)
Supplement: Table S3 — Odds ratios and 95% confidence intervals of depression according to baseline consumption of caffeinated or decaffeinated beverages, further adjusted for self-reported health status, diabetes, heart disease, and cancer. (DOCX) [file pone.0094715.s003.docx]

**Table S3. Odds ratios** ^a^ **and 95% confidence intervals of depression according to baseline consumption of caffeinated or decaffeinated beverages, further adjusted for self-reported health status, diabetes, heart disease, and cancer**

|  | Caffeinated drinks | | |  | Decaffeinated drinks | | |
| --- | --- | --- | --- | --- | --- | --- | --- |
| Beverages | Case/Control ^b^ | OR | 95% CI |  | Case /Control | OR | 95% CI |
| Coffee (cups/day) | | | | | | | |
| None | 1178/25783 | 1.00 |  |  | 1178/25783 | 1.00 |  |
| < 1 | 770/16789 | 1.03 | 0.94-1.14 |  | 1006/21047 | 1.05 | 0.96-1.15 |
| 1 | 1072/24418 | 0.98 | 0.90-1.07 |  | 789/15877 | 1.06 | 0.96-1.16 |
| 2-3 | 3049/72917 | 0.93 | 0.86-1.00 |  | 1371/30352 | 0.97 | 0.90-1.06 |
| ≥4 | 1380/29461 | 0.94 | 0.87-1.03 |  | 372/8736 | 0.89 | 0.79-1.01 |
| *P* for trend |  | 0.03 |  |  |  | 0.01 |  |
| Soft drinks (cans/day) | | | | | | | |
| None | 985/23633 | 1.00 |  |  | 985/23633 | 1.00 |  |
| < 1 | 3929/96355 | 1.01 | 0.94-1.09 |  | 3531/78095 | 1.08 | 1.0-1.16 |
| 1 | 276/5098 | 1.19 | 1.03-1.37 |  | 252/4632 | 1.19 | 1.03-1.38 |
| 2-3 | 420/8408 | 1.09 | 0.97-1.23 |  | 429/7182 | 1.30 | 1.15-1.46 |
| ≥ 4 | 350/5533 | 1.23 | 1.08-1.40 |  | 279/4633 | 1.20 | 1.04-1.38 |
| *P* for trend |  |  | 0.0002 |  |  | 0.0004 |  |
| Iced tea (cups/day) | | | | | | | |
| None | 4299/98282 | 1.00 |  |  | 4299/98282 | 1.00 |  |
| < 1 | 2529/59555 | 0.98 | 0.93-1.03 |  | 763/15620 | 1.08 | 1.00-1.17 |
| 1 | 697/16557 | 0.94 | 0.87-1.02 |  | 276/5718 | 1.04 | 0.91-1.18 |
| 2-3 | 1295/29093 | 0.93 | 0.87-0.99 |  | 593/10905 | 1.12 | 1.02-1.22 |
| ≥ 4 | 445/9406 | 0.91 | 0.82-1.01 |  | 247/3569 | 1.32 | 1.15-1.51 |
| *P* for trend |  | 0.009 |  |  |  | <0.0001 |  |
| Hot tea (cups/day) | | | | | | | |
| None | 7246/163165 | 1.00 |  |  | 7246/163165 | 1.00 |  |
| < 1 | 1216/28851 | 0.94 | 0.89-1.01 |  | 811/16413 | 1.06 | 0.98-1.14 |
| 1 | 523/13075 | 0.90 | 0.82-0.98 |  | 400/7372 | 1.13 | 1.02-1.26 |
| 2-3 | 450/11115 | 0.89 | 0.81-0.98 |  | 289/5500 | 1.08 | 0.95-1.22 |
| ≥ 4 | 138/2596 | 1.11 | 0.93-1.32 |  | 61/985 | 1.24 | 0.95-1.61 |
| *P* for trend |  | 0.13 |  |  |  | 0.01 |  |

Abbreviations: CI, confidence interval; OR, odds ratio.

^a^ Adjusted for age at baseline, sex, race, education, marital status, smoking, alcoholic beverage intake, physical activity, body mass index, energy intake, self-reported health status, diabetes, heart disease, and cancer.

^b^ Numbers may not add up to total due to missing.
