# Supplementary material for: Sweetened Beverages, Coffee, and Tea and Depression Risk among Older US Adults
Source: PLoS One. 2014 Apr 17;9(4):e94715. doi: 10.1371/journal.pone.0094715 (PMC3990543; doi:10.1371/journal.pone.0094715)
Supplement: Table S4 — Odds ratios and 95% confidence intervals of depression according to types of sweetener added to coffee or tea, further adjusted for self-reported health status, diabetes, heart disease, and cancer. (DOCX) [file pone.0094715.s004.docx]

**Table S4. Odds ratios**^a^ **and 95% confidence intervals of depression according to types of sweetener added to coffee or tea, further adjusted for self-reported health status, diabetes, heart disease, and cancer**

|  | Overall | | | |  | | Men | | | |  | | Women | | | |
| --- | --- | --- | --- | --- | --- | --- | --- | --- | --- | --- | --- | --- | --- | --- | --- | --- |
| Sweeteners | | Case/control | OR | 95% CI | |  | | Case /Control | OR | 95% CI | |  | | Case /Control | OR | 95% CI |
| Non-drinkers | | 408/9297 | 1.00 |  | |  | | 219/6044 | 1.00 |  | |  | | 189/3253 | 1.00 |  |
| None | | 4580/118082 | 0.86 | 0.78-0.96 | |  | | 2179/71644 | 0.84 | 0.73-0.97 | |  | | 2401/46438 | 0.89 | 0.76-1.04 |
| Sugar or honey | | 2603/59917 | 0.93 | 0.84-1.04 | |  | | 1390/39768 | 0.90 | 0.77-1.04 | |  | | 1213/20149 | 0.98 | 0.83-1.15 |
| Equal or aspartame | | 1778/30199 | 1.19 | 1.06-1.33 | |  | | 805/16989 | 1.19 | 1.01-1.39 | |  | | 973/13210 | 1.20 | 1.02-1.41 |
| Saccharin or Sweet-n-Low | | 1751/31624 | 1.11 | 0.99-1.24 | |  | | 825/18430 | 1.12 | 0.96-1.30 | |  | | 926/13194 | 1.11 | 0.94-1.31 |
| Other sweeteners | | 145/2349 | 1.19 | 0.97-1.44 | |  | | 51/1216 | 1.04 | 0.76-1.42 | |  | | 94/1133 | 1.31 | 1.01-1.70 |

Abbreviations: CI, confidence interval; OR, odds ratio.

^a^ Adjusted for age at baseline, sex, race, education, marital status, smoking, alcoholic beverage intake, physical activity, body mass index, energy intake, self-reported health status, diabetes, heart disease, and cancer.
